# Supplementary material for: Optimal minimal residual disease threshold in pediatric acute myeloid leukemia: A retrospective cohort study based on the TARGET database
Source: PLoS Med. 2026 May 8;23(5):e1005088. doi: 10.1371/journal.pmed.1005088 (PMC13155632; doi:10.1371/journal.pmed.1005088)
Supplement: S1 Code — (ZIP) [file pmed.1005088.s002.zip › S2 code/PROJ8_25_tbl/PROJ8_25_tbl_0.pdf]

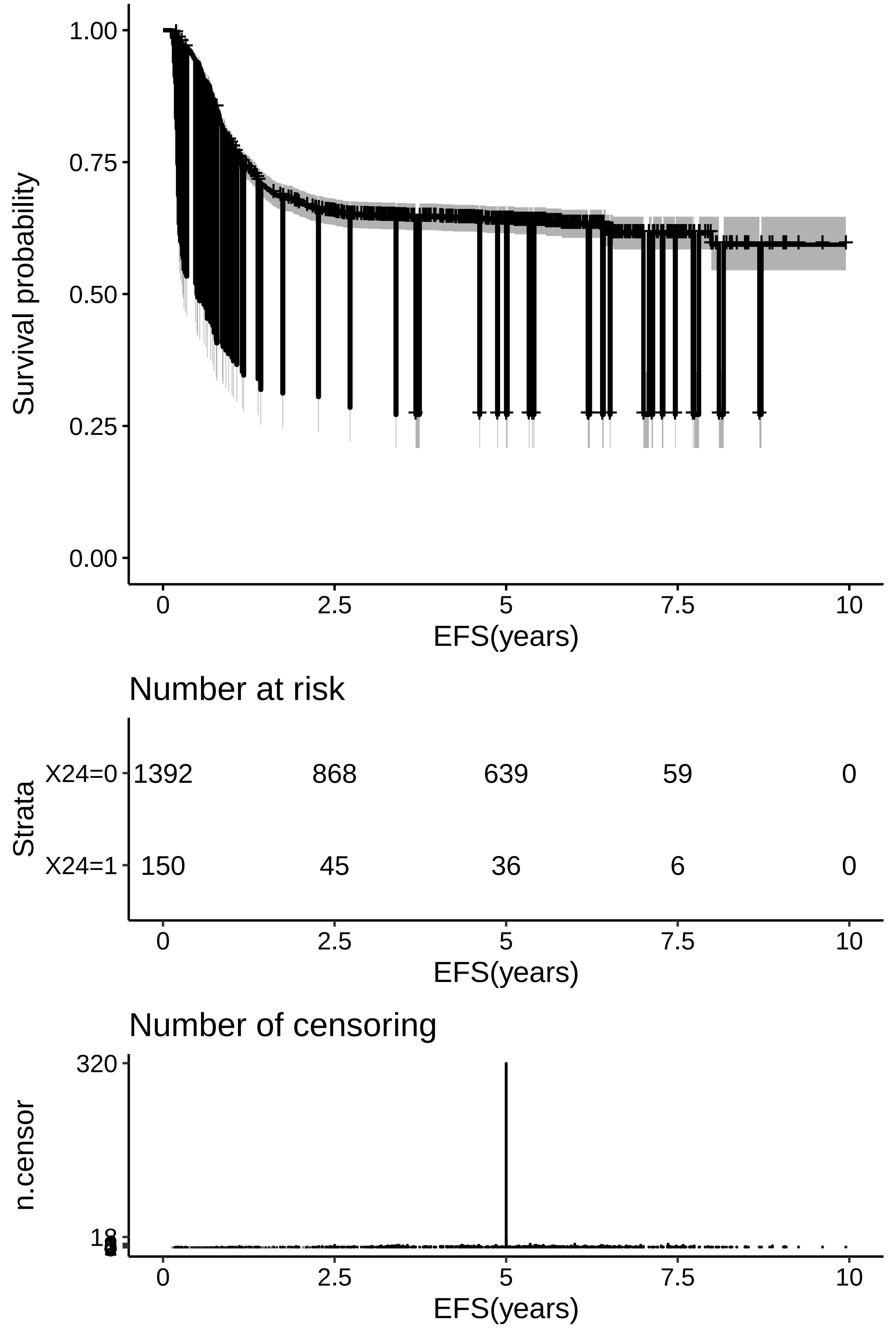
1.00
0.75
0.50
0.25
0.00
Survival probability
0
2.5
5
7.5
10
EFS(years)
Number at risk
Strata
X24=0 1392 868 639 59 0
X24=1 150 45 36 6 0
0
2.5
5
7.5
10
EFS(years)
Number of censoring
320
n.censor
0
2.5
5
7.5
10
EFS(years)
